# Clinicopathological and prognostic significance of osteopontin expression in patients with prostate cancer: a systematic review and meta-analysis

**DOI:** 10.1042/BSR20203531

**Published:** 2021-08-05

**Authors:** Anze Yu, Kai Guo, Qilin Qin, Changsheng Xing, Xiongbing Zu

**Affiliations:** 1 Department of Urology, Xiangya Hospital, Central South University, Changsha, Hunan, China; 2 Xiangya School of Medicine, Central South University, Changsha, Hunan, China; 3 Center for Inflammation and Epigenetics, Houston Methodist Research Institute, Houston, TX, U.S.A.

**Keywords:** biomarker, Osteopontin, prognosis, prostate cancer, survival

## Abstract

**Background:** Evaluation of the feasibility for osteopontin (OPN) to serve as a biomarker in the prognosis and clinical-pathological features of prostate cancer (PCA) patients.

**Methods:** The original publications related to OPN and PCA were comprehensively searched in the online databases, including PubMed, Embase, Cochrane Library, Web of Science, Medline, Wanfang and China National Knowledge Infrastructure up to August 2019. Results were analyzed by Revman 5.3 and Stata 12.0.

**Results:** A total of 21 studies were included in the analysis and the result showed that the positive OPN expression group had a lower overall survival than the negative expression group (univariate: hazards ratio (HR) = 2.32, 95% confidence interval (95% CI) [1.74, 3.10], multivariate: HR = 2.41, 95% CI [1.63, 3.57]) and a lower biochemical relapse-free survival than the negative group (univariate: HR = 1.42, 95% CI [0.92, 2.17], multivariate: HR = 1.61, 95% CI [1.39, 1.87]). In addition, there was a higher expression level of OPN in PCA tissues than in normal prostate tissues (OR = 46.55, 95% CI [12.85, 168.59], *P*<0.00001) and benign prostatic hyperplasia (BPH) tissues (OR = 11.07, 95% CI [3.43, 35.75], *P*<0.0001). Moreover, OPN positive expression was also related to high Gleason score (OR = 2.64, 95% CI [1.49, 4.70], *P*=0.0009), high TNM stage (OR = 3.15, 95% CI [1.60, 6.20, *P*=0.0009), high Whitmore–Jewett stage (OR = 2.53, 95% CI [1.06, 6.03], *P*=0.04), high lymph node (OR = 3.69, 95% CI [1.88, 7.23], *P*=0.0001), and distant metastasis (OR = 8.10, 95% CI [2.94, 22.35], *P*=0.01). There was no difference observed in the differentiation of PCA (OR = 1.79, 95% CI [0.39, 8.33], *P*=0.46).

**Conclusion:** OPN could be recognized as a promising diagnostic and prognostic biomarker for PCA patients.

## Introduction

Prostate cancer (PCA) is the second most common malignancy in males, with an increasing risk in aged population [1,2]. Current major clinical diagnostic methods for PCA include prostate-specific antigen (PSA) level test, trans-anal prostate ultrasound, prostate magnetic resonance, and needle biopsy [3–7]. Although the elevation of PSA is usually recognized as an indicator of PCA in clinical diagnosis, sometimes its production can also be induced by benign prostatic hyperplasia (BPH) or inflammation. Furthermore, the expression of PSA does not increase in some highly differentiated PCA cases [8]. Therefore, it will be critical to discover novel secretory biomarkers, which are directly associated with the clinical pathological features of PCA, to enable its convenient diagnosis and prognosis.

Osteopontin (OPN), as one of the major bone matrix proteins, is widely distributed across organs and tissues, and plays an important role in modulating inflammation and tumor responses [9]. As a cytokine homologous to matrix proteins, OPN is critical in regulating the adhesion between cells and extracellular matrix, mineralization and reconstruction of bones, cell migration, and movement [10]. OPN can also promote the cell proliferation and differentiation, long-term survival and invasion in a variety of tumors such as non-small cell lung carcinoma, prostate neoplasm, hepatic neoplasm, breast neoplasm, malignant melanoma, and colorectal neoplasm [11–17]. Consistently, an increased expression level of OPN has been observed in the peripheral blood of the patients with multiple types of tumors, compared with healthy donors, and it exhibits a positive correlation with the invasiveness and malignancy of these cancers [10].

Although OPN has the potential to serve as a novel biomarker for the diagnosis of PCA and an indicator of the level of malignancy, unfortunately, it still lacks large-scale clinical trials and a systematic analysis. Therefore, we conduct this systemic review and meta-analysis to evaluate the possibility of OPN as a biomarker for the diagnosis, malignancy, and prognosis of PCA patients.

## Materials and methods

### The criteria of inclusion

- All the studies were case-controlled or randomized-controlled trials.
- None of the patients or healthy donors ever received any radiotherapy, chemotherapy, or hormone therapy before sampling.
- All the original clinical pathological data can be obtained directly.
- The detection of OPN expression were based on the pathological examination to determine the presence of PCA tissue, rather than speculating the presence of PCA by PSA, MRI, CT, or Doppler ultrasound.
- The best data quality were selected in duplicated studies.

### The criteria of exclusion

- Animal or cell studies.
- Case report, review, meta-analysis.
- Insufficient data from original studies.

### Search strategies

The literatures were comprehensively searched in the online databases, including PubMed, Embase, Cochrane Library, Web of Science, Medline, Wanfang database, and China National Knowledge Infrastructure up to August 2019. The Clinicaltrial.gov was also searched. All the studies or clinical trials were restricted to *Homo sapiens*, but not by time, race, data source, language, age, or publication status. The following MeSH searching terms were used: (osteopontin) or (Sialoprotein 1) or (Secreted Phosphoprotein 1) or (Bone Sialoprotein 1) or (Sialoprotein 1, Bone) or (Bone Sialoprotein I) or (Sialoprotein I, Bone) or (Uropontin) AND (Prostate Neoplasms) or (Neoplasms, Prostate) or (Neoplasm, Prostate) or (Prostate Neoplasm) or (Neoplasms, Prostatic) or (Prostate Cancer) or (Prostate Cancers). Our search strategy adjusted according to different databases. Meta-analysis was performed in accordance with the Preferred Reporting Items for Systematic Review and Meta-Analysis (PRISMA) Statement.

### Quality evaluation of the included studies

We used the Newcastle–Ottawa quality assessment scale of case control studies (NOS) as the method to assess the quality of included studies. All the studies have a score range from 6 to 9, which proved that they are suitable for the inclusion.

### Data extraction and statistical analysis

All the included articles were full-text reviewed and the data were extracted by two reviewers independently. The methodological differences of classification were solved after discussion by the whole authors. The extracted data were dichotomous and analyzed by software Revman 5.3 and Stata 12.0. The results of the analyses were shown by the odds ratio (OR) and 95% confidence interval (95% CI).

Sensitivity analysis was performed to rule out the impact of a specific study when the heterogeneity of the results was large (*P*<0.1 or *I*^2^> 50%). Finally, all the included articles were tested for publication bias through Begg’s test and risk of bias summary.

## Results

### Characteristics of the included literatures

**Figure 1:**
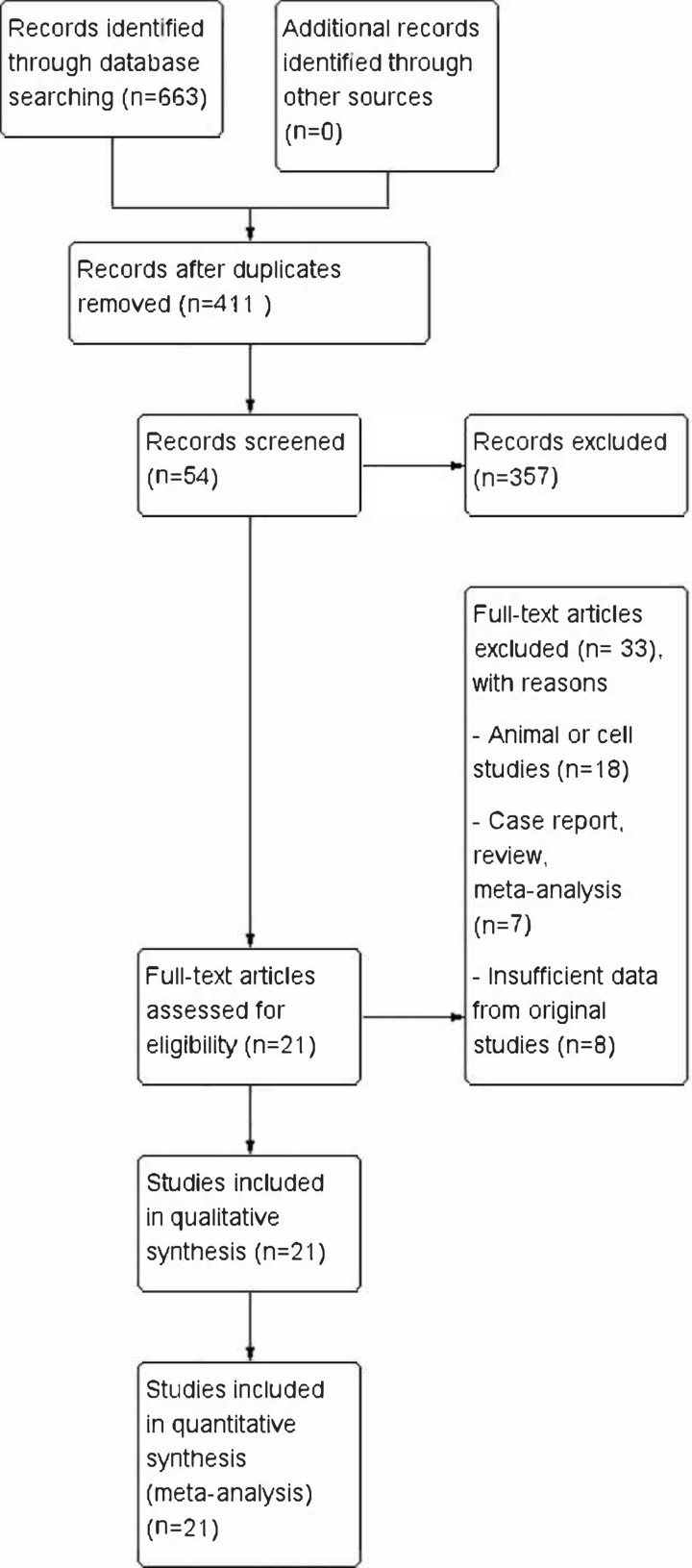
Flowchart of the included studies for meta-analysis A total of 663 studies were identified, and 252 studies were excluded because of duplication. A total of 357 studies were excluded after reading the abstract because of not related. A total of 54 possible studies were full-text reviewed and 33 studies were excluded because of animal or cell studies (*n*=18), case report, meta-analysis and review (*n*=7), insufficient data (*n*=6). Finally, 21 studies were included for quantitative analysis.

A total of 663 studies were searched and identified, including 119 from Pubmed, 301 from Embase, 20 from CNKI, 15 from Wanfang database, 4 from Cochrane Library, 120 from Google Scholar, 80 from Web of Science, and 4 from Clinicaltrial.gov. Next, 252 studies were excluded for duplication. The rest of studies were carefully examined on Title, Abstract, and Keywords, and 357 were excluded because they did not match the subject of this meta-analysis. The full text of remaining 54 studies were carefully reviewed and 33 of them were excluded as animal or cell studies (*n*=18), case report, review, meta-analysis (*n*=7), and insufficient data from original studies (*n*=8) (Figure 1). As a result, 21 studies were included for the quality evaluation through the Newcastle–Ottawa scale (NOS). The details of the included studies and the NOS score of each study are shown in Tables 1 and 2.

**Table 1:** Characteristics of the included studies for pathological parameters

Study	Year	Country	PCA (age)	BPH (age)	Normal prostate (age)	Method (thickness)	Definition of OPN positive	Indicator reported	Antibody source	NOS score
Tatina et al. [38]	2011	Brazil	29 (52–79)	18 (62–87)	30 (18–36)	Western blot	Have band	ABCFG	Invitrogen	9
Wang et al. [39]	2016	China	80 (55–85)	——	80 (55–85)	IHC (unclear)	>1 points (IHC score)	AEFG	Zymed	7
Caruso et al. [26]	2008	U.S.A.	103	——	——	IHC (unclear)	intensity > 1	C	R&D	7
Wu et al. [40]	2005	China	46 (53–70)	12 (<40)	6 (<40)	IHC (unclear)	>1 points (IHC score)	ABCEF	Chemicon	9
Tilli et al. [41]	2012	Brazil	40 (41–74)	30 (58–87)	——	IHC (4 μm) and RT-PCR	[OPNa+ OPNb+ OPNc] >29.34/1.75/9.0	BCD	Gallus Immunotech	7
Zheng et al. [42]	2014	China	80 (55–86)	35 (55–82)	35 (55–82)	IHC (4 μm)	>1 points (IHC score)	BCDG	Abbiotec	7
Flajollet et al. [43]	2011	France	35 (63)	——	——	IHC (4 μm)	>10% cells stained	C	Santa Cruz	7
Yu et al. [44]	2010	China	46 (44–70)	10 (<40)	6 (<40)	IHC (4 μm)	>1 points (IHC score)	ABCEF	Chemicon	9
Xu et al. [45]	2018	China	100 (54–87)	——	——	IHC (unclear)	>1 points (IHC score)	CDH	Linghan Shanghai	7
Wang et al. [46]	2006	China	52 (47–83)	51 (42–81)	50 (44–79)	IHC (4 μm) and ELISA	——	H	R&D	8
Tozawa et al. [18]	1999	Japan	34	12	——	IHC (unclear)	>10% cells stained	BEF	ABC	7
Forootan et al. [32]	2006	U.K.	70 (73)	36 (67.5)	10	IHC (unclear)	>10% cells stained	ABC	R&D	9
Xia et al. [47]	2012	China	108 (60–78)	100 (57–82)	——	IHC (4um)	>1 points (IHC score)	BFG	Unknown	7
Wei et al. [48]	2018	China	46 (59–86)	29	——	IHC (unclear)	>1 points (IHC score)	BCDH	Biosin	7

Abbreviations: IHC, immunohistochemistry; ELISA, enzyme-linked immunosorbent assay. IHC sore, the unstained, weakly stained, moderately stained and strongly stained cells were given 0, 1, 2, 3 scores, separately. Indicator reported: A: OPN with prostate cancer tissues and normal prostate tissues; B: OPN with prostate cancer tissues and benign prostate hyperplasia tissues; C: OPN with Gleason score of prostate cancer tissues; D: OPN with clinical TNM stage of prostate cancer tissues; E: OPN with clinical Whitmore stage of prostate cancer tissues; F: OPN with the differentiation of prostate cancer tissues; G: OPN with lymph node metastatic of prostate cancer tissues H: OPN with organ or bone metastatic prostate cancer tissues.

**Table 2:** Characteristics of the included studies for survival analysis

Study	Year	Country	Cancer types	patients	Median age [Range] (years)	Follow-up time	Counting Method	Cut-off value (positive/high)	Survival outcomes	Source of data	NOS score
Aksoy et al. [49]	2017	Turkey	mCRPC	30	——	NR	IHC	A > 50	OS (U), OS (M)	curve+direct	6
Bhattacharya et al. [50]	2019	U.K.	LPC	218	——	85 months (median)	IHC	Median scores	BRFS (U), BRFS (M) DMFS (U)	direct	7
Dayyani et al. [14]	2016	U.S.A.	mADPC	66	72 [57, 93]	1.4–2.6 years	ELISA	ln(OPN) > 11.9	PFS (U), PFS (M)	direct	7
Forootan et al. [32]	2006	U.K.	PA	70	73	0–80 months	IHC	A > 30	OS (U)	curve	6
Hotte et al. [51]	2002	Canada	HRPC	100	73 [50–86]	0–26 months	ELISA	>115 ng/ml	OS (U), OS (M)	curve+direct	8
Ramankulov et al. [52]	2007	Germany	PC	90	——	2.7–88.4 months	ELISA	>1099 μg/l	DSS (U), DSS (M)	direct	8
Vergis et al. [53]	2008	U.K.	LPC	I: 201 II: 285	I: 67 [50, 80] II: 61 [45–78]	——	IHC	A > 0	BRFS (U), BRFS (M)	direct	7

Abbreviations: A, percentage of positive cells; BRFS, biochemical relapse-free survival; DMFS, distant metastasis-free survival; DSS, disease-specific survival; ELISA, enzyme-linked immunosorbent assay; HRPC, hormone-refractory prostate carcinoma; IHC, immunohistochemistry; LPC, localized prostate cancer; M, multivariate analysis; mADPC, metastatic androgen-dependent prostate cancer; mCRPC, metastatic castrate-resistant prostate cancer; OS, overall survival; PA, prostate adenocarcinoma; PC, prostate cancer; PFS, progression-free survival; U, univariate analysis. Cohort I: radiotherapy cohort, Cohort II: radical prostatectomy cohort.

### OPN expression and overall survival

**Figure 2:**
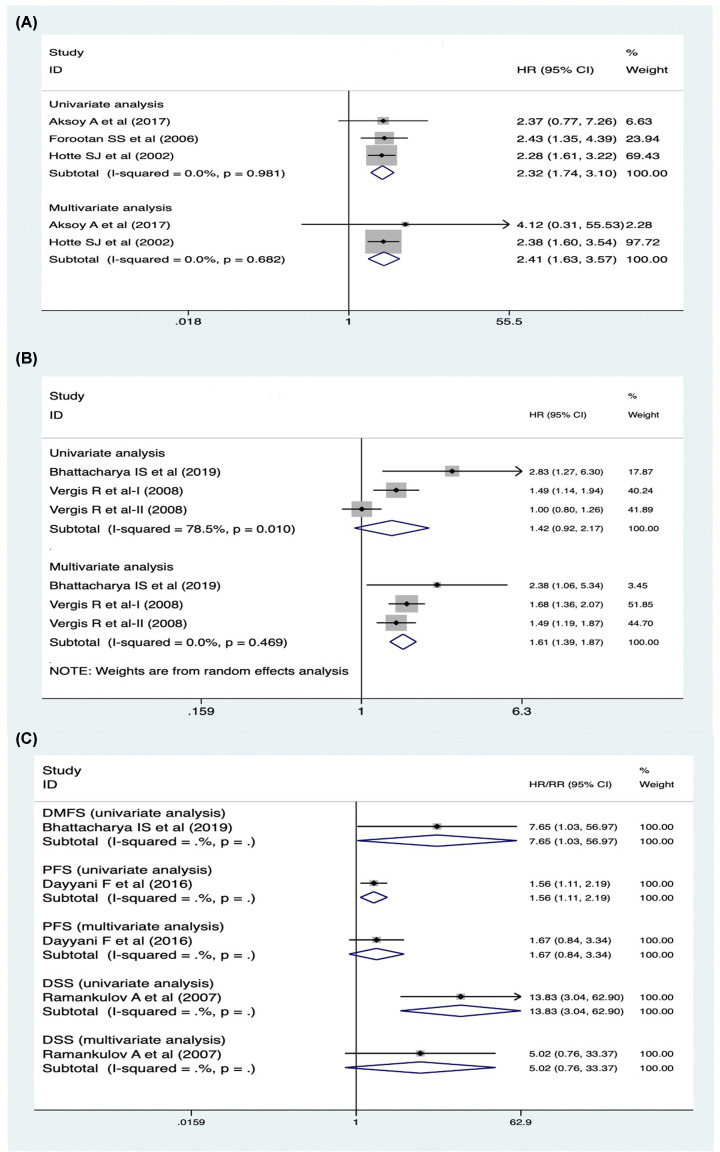
The forest plot of the expression of OPN and survival analysis (**A**) The relationship of OPN expression and overall survival (both univariate and multivariate analyses) and the result showed that OPN positive group had a lower overall survival than the negative group. (**B**) The relationship of OPN expression and biochemical relapse-free survival (both univariate and multivariate analyses) and the result showed that OPN positive group had a lower biochemical relapse-free survival than the negative group. (**C**) The relationship of OPN expression and other kind of survival analysis (Abbreviations: DMFS, distant metastasis-free survival; DSS, disease-specific survival; PFS, progression-free survival).

In the overall survival analysis, three univariate studies and three multivariate studies were analyzed separately. The univariate studies had no heterogeneity (*P*=0.981, *I^2^* = 0%), so a fixed-effects model was used and showed that the OPN positive group had a lower overall survival than the negative group (hazards ratio (HR) = 2.32, 95% CI [1.74, 3.10], *P*<0.05). Besides, the multivariate studies also had no heterogeneity (*P*=0.682, *I^2^* = 0%), so the fixed-effects model was used and presented the same result (HR = 2.41, 95% CI [1.63, 3.57], *P*<0.05) (Figure 2A). The data was stable and reliable(Figure 5A). In conclusion, the positive expression of OPN is not conducive to the overall survival in PCA patients.

### OPN expression and biochemical relapse-free survival

Biochemical relapse of PCA stands for the re-elevation of PSA level in prostate cancer patients underwent clinical therapy. A total of three studies, including four univariate analyses and four multivariate analyses, were selected for evaluating the association of OPN expression and biochemical relapse-free survival. The univariate studies had a high heterogeneity (*P*=0.010, *I^2^* = 78.5%), so the random-effects model was used and showed that the OPN positive group had a lower biochemical relapse-free survival than the negative group (HR = 1.42, 95% CI [0.92, 2.17], *P*<0.05). Sensitivity analysis showed that the result was stable(Figure 5B-C). In addition, the multivariate studies had no heterogeneity (*P*=0.469, *I^2^* = 0%), so the fixed-effects model was used and the same result (HR = 1.61, 95% CI [1.39, 1.87], *P*<0.05) was observed (Figure 2B).

### OPN expression and other survival studies

Except the overall survival and biochemical relapse-free survival analyses, some studies also mentioned the OPN expression with distant metastasis-free survival, progression-free survival, and disease-specific survival. Although these studies all showed that OPN positive group had a lower survival rate (Figure 2C), respectively, the results were not conclusive in our meta-analysis due to the lack of relevant studies.

### OPN expression between prostate cancer tissues and normal prostate tissues

**Figure 3:**
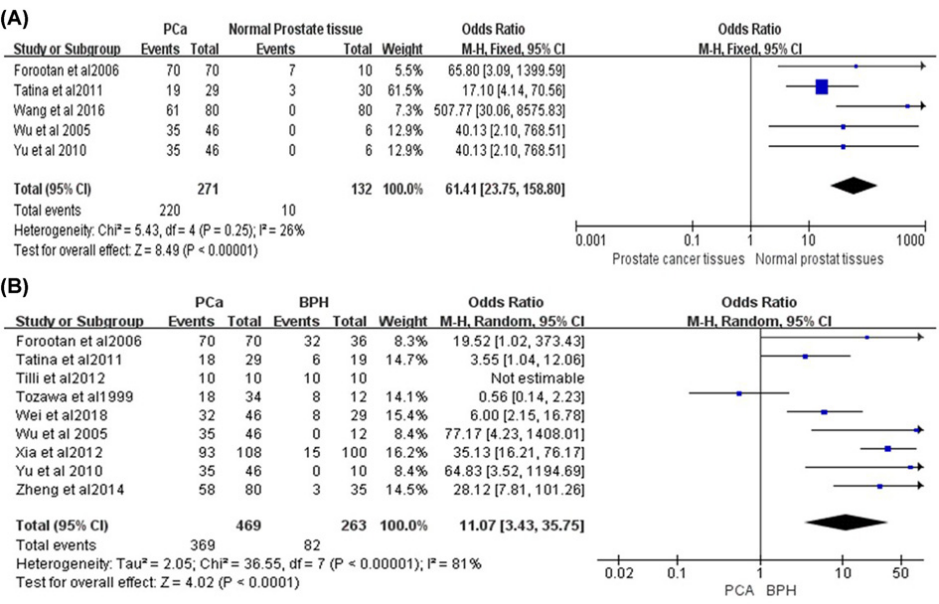
The forest plot of the expression of OPN and different prostate tissues (**A**) OPN positive expression in PCA tissues and normal prostate tissues. The result showed that OPN in PCA tissues was significantly higher than that in normal PCA tissues (OR = 46.55, 95% CI [12.85, 168.59], *P*<0.00001). (**B**) OPN positive expression in prostate cancer tissues and BPH tissues. The result showed that OPN in prostate cancer tissues was significantly higher than that in BPH tissues (OR = 11.07, 95% CI [3.43, 35.75], *P*<0.0001).

A total of five studies reported the expression of OPN between prostate cancer tissues and normal prostate tissues, including 271 PCA tissue samples and 132 normal prostate tissue samples. The heterogeneity of the obtained results is low (*P*=0.25, *I^2^* = 26%), so the fixed-effects model is adopted and showed that the expression of OPN in PCA tissues was significantly higher than that in normal prostate cancer tissues (OR = 46.55, 95% CI [12.85, 168.59], *P*<0.00001) (Figure 3A). Then, we performed the sensitivity analysis on the obtained results and excluded individual studies one by one. The heterogeneity did not change significantly, which proves that our conclusion was stable and reliable (Figure 5D). In summary, there was higher expression level of OPN in PCA tissues than in normal prostate tissues.

### OPN expression between PCA tissues and BPH

A total of nine studies reported the comparison of OPN expression in patients with PCA and BPH, including 469 PCA tissue samples and 263 BPH tissue samples. The results obtained from the analysis had a high heterogeneity (*P*<0.00001, *I^2^* = 81%), so the random-effects model was adopted. To further determine the stability and reliability of the results, we performed a sensitivity analysis of the included literatures to determine whether individual studies would have an impact on the results. And we found that the heterogeneity decreased after excluding the study of Tozawa et al. [18], with the conclusion that there was higher expression level of OPN in PCA tissues than in BPH tissues (OR = 11.07, 95% CI [3.43, 35.75], *P*<0.0001). The result was stable and acceptable (Figure 5E). In summary, the expression of OPN in PCA tissues was higher than in BPH tissues (Figure 3B).

### OPN expression with the Gleason score of PCA

**Figure 4:**
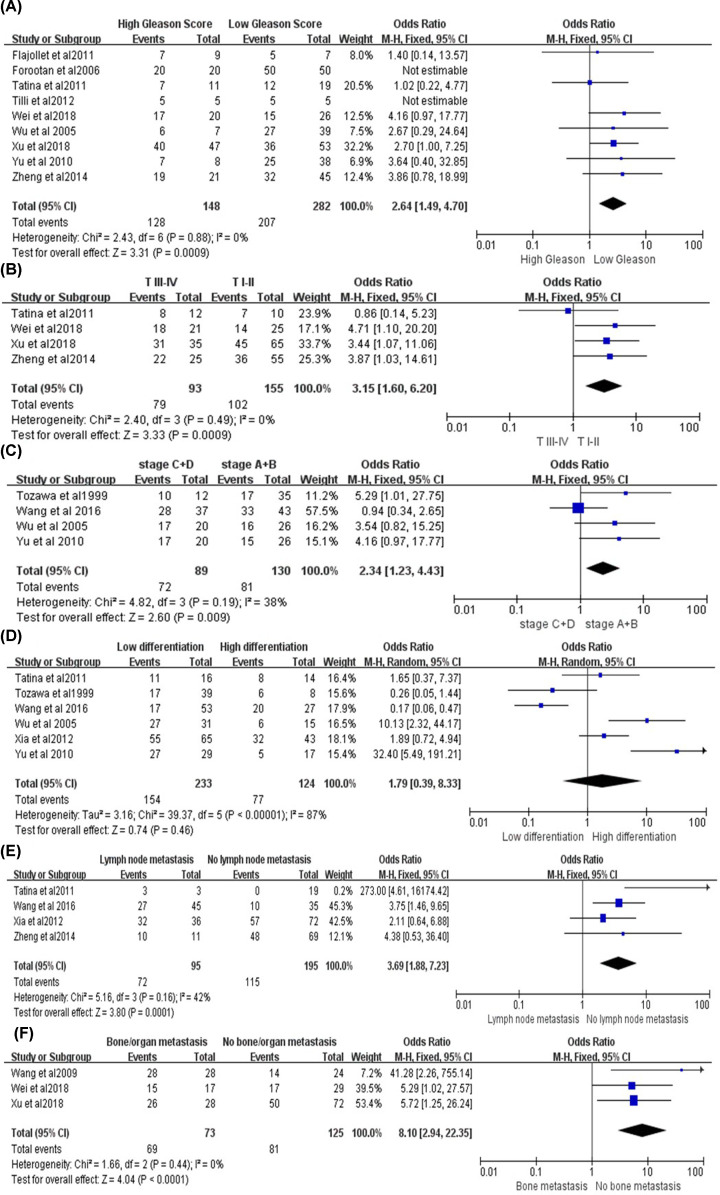
The forest plot of the expression of OPN and clinical pathological features of PCAs (**A**) The relationship between OPN positive expression and Gleason score. The result showed that the positive rate of OPN in high Gleason score patients was significantly higher than the ones with low Gleason score (OR = 2.64, 95% CI [1.49, 4.70], *P*=0.0009). (**B**) The relationship between OPN positive expression and clinical stage. The result showed that the expression level of OPN in T III–IV stage is higher than that in T I–II stage of PCA patients (OR = 3.15, 95% CI [1.60, 6.20], *P*=0.0009). (**C**) The relationship between OPN positive expression and Whitmore-Jewett stage. The expression level of OPN in Whitmore-Jewett stage C+D patients is higher than in stage A+B patients. (OR = 2.53, 95% CI [1.06, 6.03], *P*=0.04). (**D**) The relationship between OPN positive expression and the differentiation of PCA. The expression of OPN have no difference between high and low differentiation of PCA tissues. (OR = 1.79, 95% CI [0.39, 8.33], *P*=0.46). (**E**) The relationship between OPN positive expression and the lymph node metastasis. The expression of OPN is positively correlated with lymph node metastasis of PCA. (OR = 3.69, 95% CI [1.88, 7.23], *P*=0.0001). (**F**) The relationship between OPN positive expression and distant metastasis of PCA. The expression of OPN is positively correlated with distant metastasis of PCA (OR = 8.10, 95% CI [2.94, 22.35], *P*=0.01).

**Figure 5:**
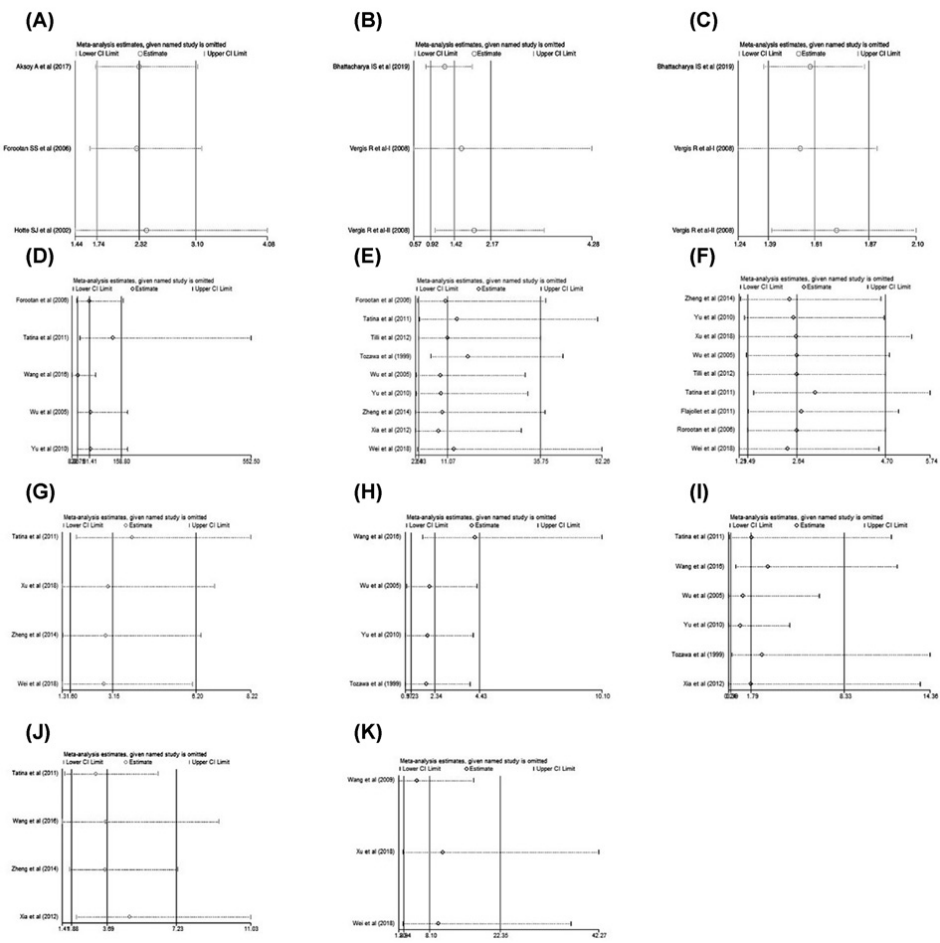
The sensitivity analysis of each analysis (**A**) The expression of OPN and overall survival (univariate analysis). (**B**) The expression of OPN and biochemical relapse-free survival (univariate analysis). (**C**) The expression of OPN and biochemical relapse-free survival (multivariate analysis). (**D**) The positive expression of OPN between PCA tissues and normal prostate tissues. (**E**) The positive expression of OPN between PCA tissues and BPH tissues. (**F**) The positive expression of OPN with Gleason score. (**G**) The positive expression of OPN with clinical stage. (**H**) The positive expression of OPN with Whitmore–Jewett stage. (**I**) The positive expression of OPN with differentiation of PCA. (**J**) The positive expression of OPN with lymph node metastasis of PCA. (**K**) The positive expression of OPN with distant metastasis of PCA.

Gleason score is a widely used histological scoring system in PCA, which is associated with the prognosis and biological behavior. A total of nine studies reported the relationship between OPN expression and the Gleason score of PCA tissues, including 148 high Gleason score samples (>8) and 282 low Gleason score samples (≤7). The result had no heterogeneity (*P*=0.88, *I^2^* = 0%), and the fixed-effects model was adopted (Figure 4A). Sensitivity analysis showed the result was stable (Figure 5F). The studies of Forootan et al. [32] and Tilli et al. [38] found that all the PCA tissues were positively stained with OPN, and they compared the Gleason score to the staining intensity of OPN. Although all PCA tissues were positive for OPN staining from their studies, the prostate cancer tissues with high Gleason score had a stronger staining when compared with the low Gleason score tissues. In conclusion, the expression level of OPN was positively correlated with the Gleason score of prostate cancer (OR = 2.64, 95% CI [1.49, 4.70], *P*=0.0009).

### OPN expression with the clinical TNM stage of PCA

A total of four studies included the relationship between OPN and the clinical TNM stage of prostate cancer, among which 93 T III–IV stage patients and 155 T I–II stage patients were reported. The results had no heterogeneity (*P*=0.49, *I^2^* = 0%), and the fixed-effects model was adopted (Figure 4B). Sensitivity analysis showed the result was stable (Figure 5G). Overall, the expression level of OPN in T III–IV stage was higher than that in T I–II stage of PCA patients (OR = 3.15, 95% CI [1.60, 6.20], *P*=0.0009).

### OPN expression with the Whitmore–Jewett stage of PCA

Whitmore–Jewett stage is the most common staging strategery in clinical practice. This staging method divides PCA into four stages (A, B, C, D) according to the degrees of infiltration. A total of four studies reported the relationship between OPN and the Whitmore–Jewett stage of PCA, including 89 stage C+D patients and 130 stage A+B patients. The results had a low heterogeneity (*P*=0.19, *I^2^* = 38%), and the fixed-effects model was used (Figure 4C). When we conducted the sensitivity analysis and excluded the studies in turn, we found that the heterogeneity did not change significantly, so the analysis results were stable (Figure 5H). Over all, the expression level of OPN in Whitmore–Jewett stage C+D patients was higher than in stage A+B patients (OR = 2.53, 95% CI [1.06, 6.03], *P*=0.04).

### OPN expression with the differentiation of PCA

A total of six studies reported the relationship between OPN and the differentiation of prostate cancer, including 233 low differentiation samples and 124 high differentiation samples. The results had a significant heterogeneity (*P*<0.00001, *I^2^* = 87%), so the random-effects model was adopted and showed that the expression of OPN had no difference between high and low differentiation of PCA tissues (OR = 1.79, 95% CI [0.39, 8.33], *P*=0.46) (Figure 4D). Then we conducted a sensitivity analysis, and after removing each study one by one, the heterogeneity remained constant, which proved that our results were reliable (Figure 5I). In conclusion, there was no significant correlation between the expression level of OPN with the differentiation of prostate cancer.

### OPN expression with the lymph node metastasis of PCA

Lymph node metastasis of tumors is often associated with poor prognosis, so the early diagnosis and judgement of lymph node metastasis is essential. A total of four studies included the relationship between the OPN and lymph node metastasis of PCA, including 290 PCA tissues. The result had a low heterogeneity (*P*=0.16, *I^2^* = 42%), so the fixed-effects model was adopted, and showed that the expression of OPN was positively correlated with lymph node metastasis of PCA (OR = 3.69, 95% CI [1.88, 7.23], *P*=0.0001) (Figure 4E). Sensitivity analysis showed the result was stable (Figure 5J).

### OPN with the distant metastasis of PCA

Distant metastasis including bone or organ metastasis are the most common forms of metastasis in patients with PCA and usually bring a poor prognosis. Three articles including 198 patients have reported the correlation between PCA distant metastasis and OPN. The results had no heterogeneity (*P*=0.44, *I^2^* = 0%), so the fixed-effects model was adopted, and showed that the expression of OPN was positively correlated with distant metastasis of PCA (OR = 8.10, 95% CI [2.94, 22.35], *P*=0.01) (Figure 4F). Sensitivity analysis showed that the result was stable (Figure 5K).

### Publication bias

**Figure 6:**
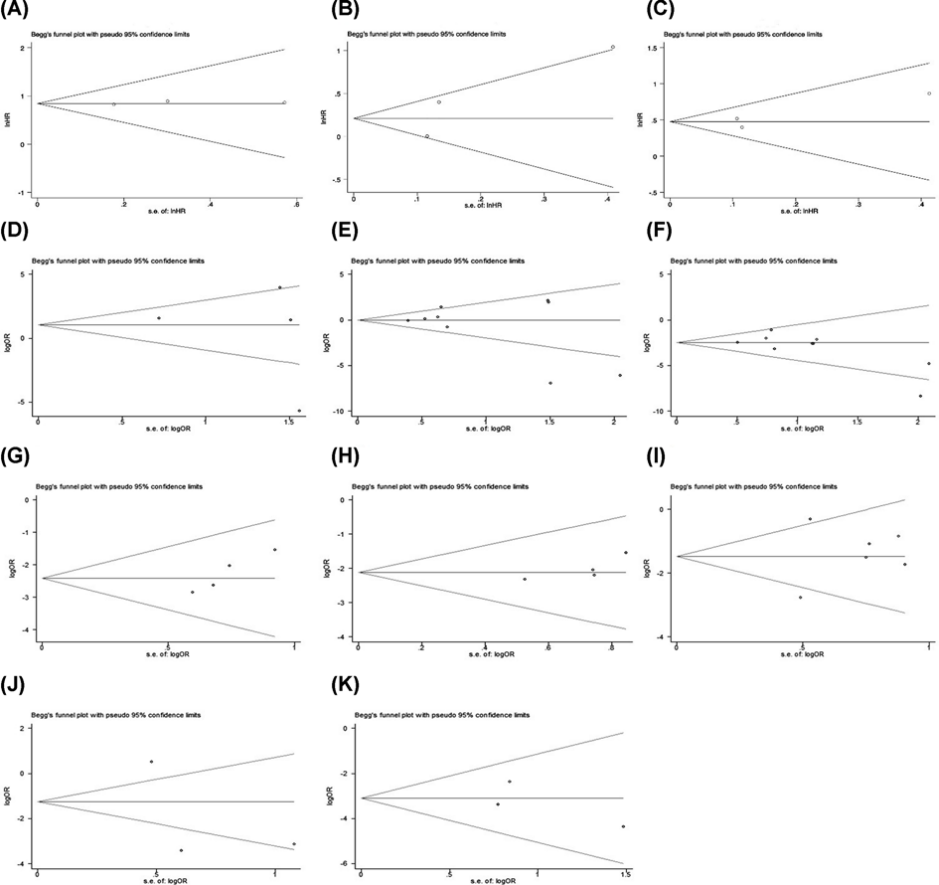
Publication bias (**A**) The expression of OPN and overall survival (univariate analysis). (**B**) The expression of OPN and biochemical relapse-free survival (univariate analysis). (**C**) The expression of OPN and biochemical relapse-free survival (multivariate analysis). (**D**) The positive expression of OPN between PCA tissues and normal prostate tissues. (**E**) The positive expression of OPN between PCA tissues and BPH tissues. (**F**) The positive expression of OPN with Gleason score. (**G**) The positive expression of OPN with clinical stage. (**H**) The positive expression of OPN with Whitmore–Jewett stage. (**I**) The positive expression of OPN with differentiation of PCA. (**J**) The positive expression of OPN with lymph node metastasis of PCA. (**K**) The positive expression of OPN with distant metastasis of PCA.

**Figure 7:**
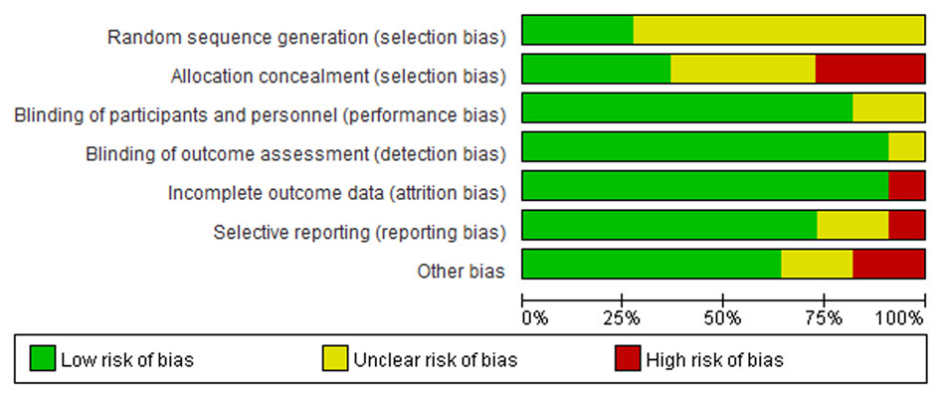
Risk of publication bias summary diagram Diagram showed the source and risk of potential bias.

After analyzing all the results, we examined whether there was publication bias in the included literatures by using Begg’s test, and the conclusions showed that there was no significant publication bias in each analysis (Figure 6), so the results were reliable and stable. Besides, we also conducted the risk of bias summary diagram to show the source of potential bias (Figure 7).

## Discussion

PCA is an epithelial malignancy with the second highest incidence rate in aged men, and the patients are usually treated with castration therapy or surgery [9,19–24]. However, there still lack of effective treatments for advanced metastatic PCA and castration-resistant PCA. OPN is a secreted phosphorylated glycoprotein which is very important in the cell proliferation and differentiation of various malignant tumors [25]. It is also involved in bone metabolism, induces angiogenesis, inhibits the killing function of immune cells in the tumor microenvironment, thereby promoting tumor invasion and metastasis [11,26,27]. Increasing studies report that OPN is associated with the proliferation and metastasis of a variety of tumors, including breast cancer, stomach cancer, PCA, liver cancer, and esophageal cancer [8,10,25,28–30].

OPN binds to its receptor integrin and regulates the migration, adhesion, and mobilization of tumor cells, which is related to the invasion and proliferation ability of multiple cancers [31]. Firstly, we investigated the expression of OPN with the survival analyses (overall survival, biochemical relapse-free survival, disease specific survival, distant metastasis-free survival, and progression-free survival) in PCA patients and all the results showed that the high expression of OPN could decrease the survival time. Meanwhile, we also analyzed the expression of OPN in different types of prostate tissues, and found that the positive expression rate of OPN in PCA tissues is higher when compared with BPH tissues and normal tissues. However, some studies also showed a different conclusion. Forootan et al. [32] showed that OPN had a high positive expression rate in BPH and normal tissues. Tilli et al.’s research showed that there was a similar level of OPN expression between PCA tissues and BPH tissues. Additionally, we analyzed the differential expression of *SPP1* between 52 normal prostate samples and 498 PCA samples in The Cancer Genome Atlas (TCGA) database. No significant difference in *SPP1* expression was detected between normal tissues and PCA tissues (log_2_FC = 0.162, FDR = 8.57E-4) (Supplementary Figure S1). This meta-analysis and TCGA database displayed the contradictory results. This discrepancy may arise from the different detection methods. The pooled analysis is compromise of four studies based on IHC method and one study based on western blot method, while the samples included in the TCGA database are based on RNA-seq data. Therefore, we need more researches and evidences to further solve this problem.

In addition, our meta-analysis compared the expression of OPN in relation to the clinical pathological parameters of PCA such as Gleason score, the clinical TNM stage, the Whitmore–Jewett stage, the differentiation, the lymph node and distant metastasis. Overall, the expression of OPN was positively correlated with the malignant degree and metastatic ability of PCA. However, comparisons showed that there was no obvious correlation between the expression of OPN and the degree of PCA differentiation. We reason that it is due to the subjectivity of different researchers for them to define the degree of differentiation, because some researchers categorized the poorly and moderately differentiated PCAs together, while others categorized the moderately and highly differentiated PCAs together. Therefore, the correlation between the positive expression rate of OPN and the differentiation of PCA was still uncertain, and more researches are needed to achieve a conclusion.

In addition to the detection of OPN expression in prostate tissue by IHC, some scholars have also detected OPN expression in blood and urine by enzyme-linked immunosorbent assay (ELISA) [11,33,34]. The results showed that the expression of OPN in PCA patients was significantly higher than that in healthy person. In addition, OPN protein expression was detected by western blot, and its mRNA expression was detected by qPCR [28,35].

OPN expression has been indicated to correlate with cancer prognosis and survival. Lindahl et al.’s [36] study has shown that OPN can serve as an inflammatory mediator to the pathogenesis of breast cancer. Feng et al.’s [37] study shows that the up-regulation of OPN is not conducive to the survival in human lung cancers. However, our study is the first meta-analysis to discuss the relationship between survival and OPN expression, and we also evaluate the expression of OPN in different prostate tissues and different clinical parameters. Meanwhile, some restrictions and limitations still exist in our study. Firstly, our meta-analysis selected the data from published literatures rather than all raw data, so our results of the analyses may deviate from the true values if the cited articles have bias. Secondly, different studies often choose various criteria for judging the expression level of OPN, which may lead to some different conclusions.

## Conclusion

The results of our meta-analysis indicate that OPN positive expression is not conducive to overall survival and biochemical relapse-free survival in PCA patients. OPN has a higher positive rate in PCA than in BPH tissues and normal tissue. In addition, the positive rate of OPN expression was positively correlated with Gleason score, TNM stage, Whitmore–Jewett stage, lymph node metastasis, and bone metastasis.

## Supplementary Material

Supplementary Figure S1

## Data Availability

The datasets used in the present study are available from published papers that have been cited in the present paper. All data are available from the corresponding author upon reasonable request.

## Abbreviations

BPH: benign prostatic hyperplasia
FDR: false discovery rate
HR: hazards ratio
NOS: Newcastle–Ottawa scale
OPN: osteopontin
PCA: prostate cancer
PSA: prostate-specific antigen
TCGA: The Cancer Genome Atlas
TNM: tumor node metastasis
95% CI: 95% confidence interval
