# Supplementary material for: Clinicopathological and prognostic significance of osteopontin expression in patients with prostate cancer: a systematic review and meta-analysis
Source: Biosci Rep. 2021 Aug 5;41(8):BSR20203531. doi: 10.1042/BSR20203531 (PMC8350436; doi:10.1042/BSR20203531)
Supplement: Supplementary Figure S1 [file BSR-2020-3531_supp.pdf]

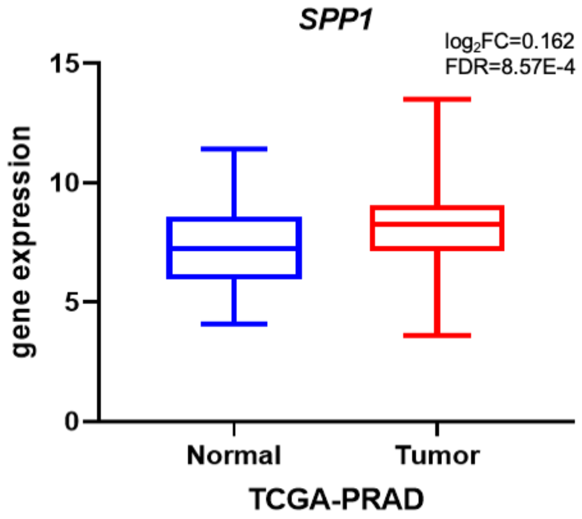

Supplementary Material

Figure S1. Box plot of *SPP1* expression between normal prostate samples and prostate cancer samples in TCGA database.
